# Reading development at the text level: an investigation of surprisal and embeddingbased text similarity effects on eyemovements in Chinese early readers

**DOI:** 10.16910/jemr.13.6.2

**Published:** 2020-09-09

**Authors:** Xi Fan, Ronan Reilly

**Affiliations:** Guangzhou Medical University, China; Maynooth University, Ireland; Maynooth International Engineering College, Fuzhou University, China

**Keywords:** Eye movements, saccades, reading development, text effect

## Abstract

This paper describes the use of semantic similarity measures based on distributed representations of words, sentences, and paragraphs (so-called “embeddings”) to assess the impact of supra-lexical factors on eye-movement data from early readers of Chinese. In addition, we used a corpus-based measure of surprisal to assess the impact of local word predictability. Eye movement data from 56 Chinese students were collected (a) in the students’ 4th grade and (b) one year later while they were in 5th grade. Results indicated that surprisal and some text similarity measures have a significant impact on the momentto- moment processing of words in reading. The paper presents an easy-to-use set of tools for linking the low-level aspects of fixation durations to a hierarchy of sentence-level and paragraph-level features that can be computed automatically. The study is the first attempt, as far as we are aware, to track the developmental trajectory of these influences in developing readers across a range of reading abilities. The similarity-based measures described here can be used (a) to provide a measure of reader sensitivity to sentence and paragraph cohesion and (b) to assess specific texts for their suitability for readers of different reading ability levels.

## Introduction

As a reader progresses through a text, depending on their reading goal, they encounter words from which they construct phrases, integrate them into larger sentential and discourse units, and use them to create an isomorphic representation of the writer’s conceptual structure. Information is acquired from words or word clusters and then integrated into conceptual units of coarser granularity, such as ideas, events, episodes, narratives. There is considerable evidence that the ongoing cognitive processes involved in reading have a direct impact on the lower-level information processing stages involved in eye movement control (see Rayner (1), Radach and Kennedy (2, 3), for overviews).

### Measures of eye movements

**Figure 1.**
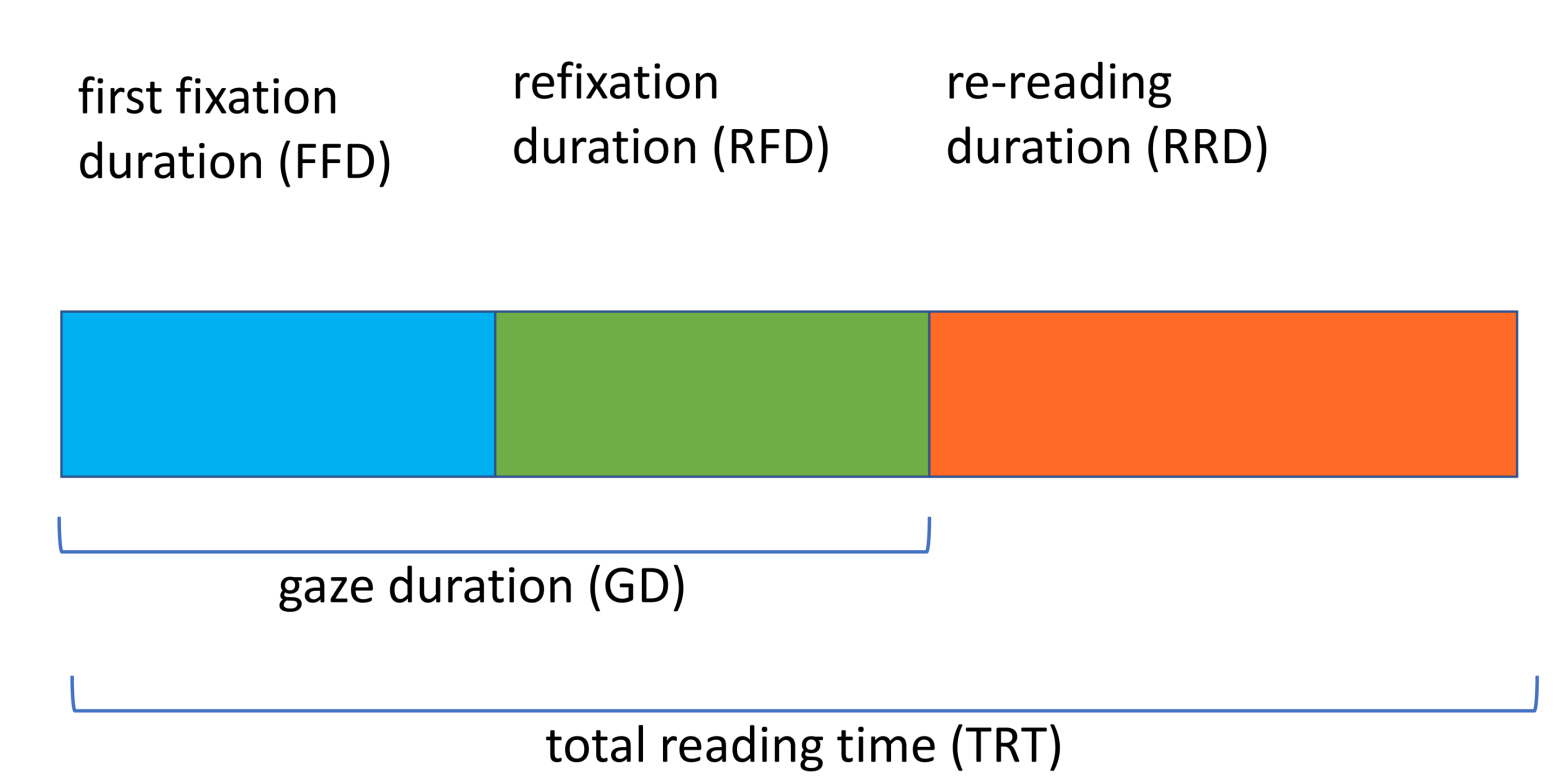
The word-viewing time decomposition used in this paper allows the separate components of first-fixation duration (FFD), refixation duration (RFD), and re-rereading duration (RFD) to be combined additively to give the total reading time on the word. The commonly used gaze duration (GD) measure is the sum of FFD and RFD.

Using eye-tracking technology, researchers can measure the eye movement characteristics of readers during reading and use the data to probe the underlying perceptual and cognitive process involved in decoding text into a meaning representation. The two relevant eye movement events in reading are ﬁxations, where the eye is relatively still, and saccades, where the eye makes ballistic movements across the text. There is a variety of metrics derived from the duration of ﬁxations that are used by reading researchers to provide insight into the time course of reading-related perceptual and cognitive processes. For example, ﬁrst ﬁxation duration (FFD) is the duration of the ﬁrst ﬁxation on a word from an incoming rightward saccade and tends to reﬂect early processing of the word. Reﬁxation duration (RFD) is the sum of subsequent ﬁxations on the word before the eye leaves it and will reﬂect processing demands related to the frequency of occurrence of a word. Re-reading duration (RRD) measures any subsequent viewing time on the word after the eyes have left it and tends to measure the diﬃculty the reader has in integrating the word into a larger meaning representation. Another commonly used measure of word processing is gaze duration, which is the sum of FFD and RFD (1). Figure 1 is a schematic representation of the relationship between these word-based viewing time measures. There are other duration-based measures that aim to capture the processing of larger text regions. However, since our focus is primarily on the earlier stages of word processing, our primary focus will be on the FFD, RFD, and RRD measures (see Radach and Kennedy (2), for a further discussion of these measures).

### Measures of text relatedness

Because of the structural limitations of the eye, the pick-up of word information in reading is a processing bottleneck, but its inherent limitation also presents us with the opportunity to observe the impact of higher level factors relating to sentence structure and meaning on the deployment of this constrained resource. It is possible, therefore, to observe both the impact of processing the current word, but also of prior context through variations in a variety of eye movement parameters. A major challenge in this area of research is to develop quantitative measures of text complexity at a variety of levels that can be used to predict eye movement behaviour. Ideally, one would like to model faithfully the grammatical and conceptual knowledge that the reader is bringing to bear on the task and use it to predict reading behaviour. Levy (4) proposed the concept of surprisal as a quantitative measure of the cognitive cost required to process a word in a sentence. Surprisal is a text metric that captures how predictable a word is, given the context of preceding words. Substantial progress has been made in developing the measure with the use of conditional probability distributions over interpretations (5). A number of easy-to-calculate proxies for surprisal can be derived from various features of large text corpora. If the corpus is large enough, such as is the case with the Google and Microsoft n-gram corpora (6, 7), then we can obtain usable n-gram frequency counts from unigrams (single words) through to 5-grams (e.g., an n-gram is a sequence of N words, a 5-gram is a ﬁve-word sequence). The n-gram frequency counts can be used to calculate surprisal values that can act as approximations to both syntactic and to a lesser extent semantic expectations. These in turn can be assessed as predictors of various eye movement parameters.

While surprisal may capture some of the dynamic local constraints on the reader, clearly there are other things going on during reading. For example, if the reader is dealing with an extended text, he or she has the challenge of integrating meaning across sentences. Depending on the coherence of the text, this can prove more or less diﬃcult. Moreover, the current sentence stands in some relation to the text as a whole to the degree that it’s more or less central to the theme. A pioneering attempt to quantify global context eﬀects on text reading was reported by Pynte et al. (8, 9) using Latent Semantic Analysis (LSA). LSA (10) is a theory and method for analysing documents to ﬁnd the underlying meaning or concepts inherent in the documents. The central idea is that the aggregate of all the word contexts in which a given word does and does not appear provides a set of mutual constraints that largely determines the similarity of meaning of words and sets of words to each other. LSA is one of the most commonly used methods for word meaning representation. However, in recent years neural-network language “embeddings” have received increasing attention (1, 12, 13, 14, 15). Furthermore, neural-network derived language embeddings tend to have better performance than LSA on very large training corpora (16).

**Figure 2.**
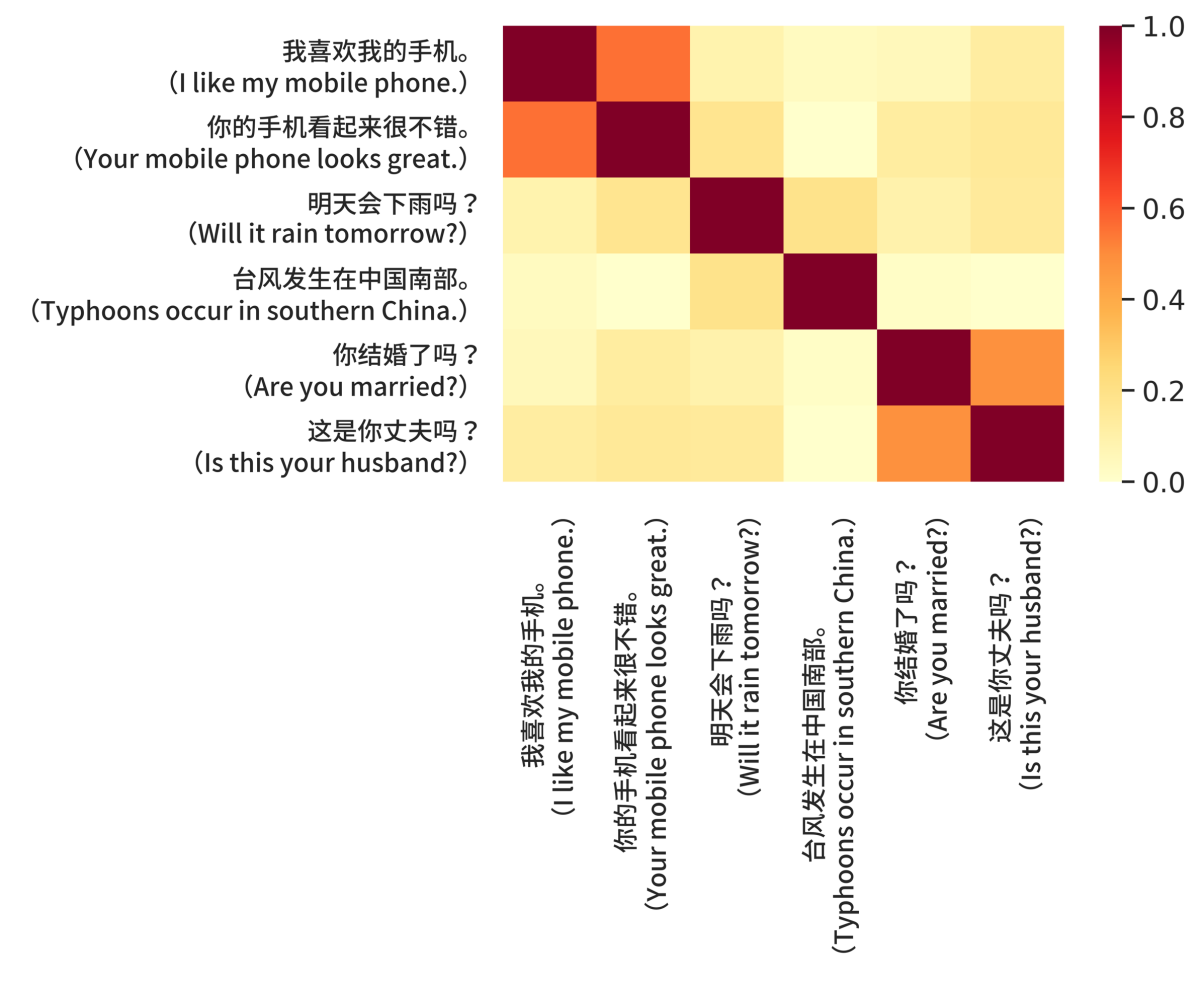
Sentence similarity scores using embeddings from the Chinese version of the universal sentence encoder (19). Note that the darker the cell, the greater the sentence similarity. Hence, the diagonal cells are the darkest since they represent self-similarity. The sentence pairs relating to phones and marriage are considered most similar when compared to the pair relating to weather. This may be due to the absence of overlapping words and word stems in the latter two sentences.

Neural-network language embeddings exploit statistical properties of text structure to embed text (words, sentences or paragraphs) into numerical vectors of a ﬁxed number of dimensions (usually between 100 and 500), with more dimensions supporting more nuanced discrimination between meanings. The intuition behind the embedding is that it represents text based on its lexical context accumulated over many millions of instances. Consequently, text appearing in similar contexts will have similar embeddings. Embeddings allow us to easily compute semantic similarity between two texts. A typical way of calculating semantic similarity of language items is to measure the cosine of the angle between the high-dimensional vectors representing the language items; the larger the cosine value, the greater the similarity. A signiﬁcant recent trend has been the development of so-called universal embeddings (17, 18). Universal embeddings are trained on a variety of data sources and use text classiﬁcation, semantic similarity, clustering, and other natural language tasks to improve their performance by forcing them to incorporate more general word and sentence features. Google’s Universal Sentence Encoder is one example of this approach (19, 20). Figure 2 is intended to show that sentence embeddings can be trivially used to compute sentence level semantic similarity scores that achieve excellent performance on the semantic textual similarity (STS) benchmark (1). Google’s Universal Sentence Encoder is available on TensorFlow Hub (https://tfhub.dev/google/universalsentence-encoder). The website provides easy-to-use code templates that can be used to encode words, sentences or paragraphs into high dimensional vector embeddings. This then allows the straightforward calculation of similarity among words, sentences and paragraphs.

### Previous research on reading development

In contrast to the substantial literature on adult reading, the number of research publications on eye movements in developing readers is still quite limited (21, 22). Much of the groundwork was laid by a set of pioneering studies of text reading (23, 24, 25). These early studies examined elementary school students across various grades, with quantitative analyses mostly restricted to global parameters such as average ﬁxation duration, number of ﬁxations per 100 words, and overall regression frequency. Not surprisingly, this work documented a steady reduction of ﬁxation durations and the number of ﬁxations from grade to grade, whereas the decrease was less pronounced for the proportion of regressive saccades. As Rayner (26) has pointed out, these developmental changes in eye movements and the analogous diﬀerences in the eye movement patterns of readers of diﬀering ability are not the cause of diﬀerences in reading ﬂuency. Rather, longer ﬁxation durations, shorter saccades, and more regressions all reﬂect the fact that reading involves the coordination of many diﬀerent perceptual and cognitive process and that this coordination takes time to develop and automatise and some beginning readers are more adept at it than others.

McConkie et al (25) pioneered the analysis of children’s eye movements at the word level, including fine grained analyses of saccade landing sites within words, and the first quantitative analyses of relations between eye movement parameters and psychometric reading assessments. This was also one of the first studies in which the now common decomposition of viewing times into initial fixation duration, gaze duration and re-reading time was applied to research on developing readers.

Most current studies on eye movements in developing readers have used experimental designs to address speciﬁc research questions, usually comparing children with adults or readers at diﬀerent stages of their development. This work has usually focused on sub-lexical and lexical components of the reading process, such as letter recognition within the perceptual span (1, 27) or eﬀects of word length and frequency (28, 29, 30, 31, 32, 33). In contrast, higher level, post-lexical processing beyond the word level has received very limited attention. The few exceptions include work on semantic plausibility (34, 35), syntactic ambiguity (33) and local comprehension monitoring (36). Van der Schoot et al. (37) examined global text comprehension using a narrative inconsistency task that allowed individual diﬀerences to be assessed in terms of the development and updating of a coherent mental representation of a text passage.

### The present study

Complementing this previous experimental work, the present paper will examine supra-lexical effects using surprisal and embedding similarities in a large corpus of eye movement data collected in a longitudinal study of Chinese elementary school students. In the study, the total accumulated time spent on a word during reading was partitioned into three non-overlapping components: first fixation duration (FFD), re-fixation duration (RFD), and the remainder of any viewing time on the word, which will be refer to as re-reading duration (RRD). The central hypothesis of the study is that eye movement metrics are sensitive to surprisal and supra-lexical semantic similarity measures. We should also see developmental changes in sensitivity to these text measures. With respect to local context effects as expressed in surprisal measures, the hypothesis is that surprisal measure will have a stronger effect in the early viewing time phase as it quantifies how predictable a word is, while supra-lexical semantic similarity measures will have a stronger influence in the later viewing time phase as they relate to higher-order semantic features of the sentence and paragraph. Furthermore, over the course of development there should be a gradual increase in the sensitivity of readers to such influences. Therefore, it is predicted that there is a greater likelihood of surprisal effects in 5th graders rather than 4th graders, with a gradual increase as they become more skilled readers. Similarly, with respect to global contextual influences it would be expected to see a growing sensitivity to sentence coherence as a child increased their reading proficiency.

## Methods

### Participants

The study involved two large-scale longitudinal data collections at Huilai Yingnei primary school in Guangdong Province, China. Data was collected from the same cohort of students in 2017 and 2018. Raven’s Standard Reasoning Test, normed for China (38), and the Literacy Test for the Primary School Students (39) were administered to all 768 grade 4 students attending the school as intelligence and literacy tests, respectively. The Literacy Test for the Primary School Students is a standardised test to measure pupil’s vocabulary size which has high reliability and validity, both of which are 0.8. It has been used in research to assess pupil’s reading ability, since how well a child can read is correlated with the size of their vocabulary (40). The results of the Chinese language end-of-term exam, which was administered by the school just prior to the experiment, was also used as a participant selection criterion. The exam is used as an indicator of participants’ reading comprehension as it includes paragraph reading and writing tasks. Fifty-nine participants of diﬀerent reading ability were chosen from the experiment described for the analysis in this paper. Each experiment took between 30 and 40 minutes per participant.

The "poor" reader group came from 20 students with the lowest literacy test scores and below average Chinese term exam scores. The "good" reader group came from 19 students of similar age and intelligence level but with the highest literacy test scores and above average Chinese term exam scores. The "average" reader group comprised 20 students of similar age and intelligence level but with literacy test scores within +/−0.3 standard deviation (SD) of the mean and with Chinese term exam scores within+/−0.5 SD of the mean. Finally, all the students in the study had normal or corrected vision and had not participated in any prior eye movement studies or similar reading tests.

### Materials

Reading material were a translation of age appropriate short stories from the Florida Assessment for Instruction in Reading (FAIR) toolkit (41). Five and six stories were used in the first and second data collections, respectively. Each story was presented in multiple paragraphs (3-4 paragraphs), with each paragraph consisting of 5-7 lines (42). Word length ranged from 1 to 5 characters with a total of 4610 characters, 592 unique. Stories were presented on the screen one by one. Each story was followed by three comprehension questions. Paragraphs were displayed in black on a light grey background using a 19.5-inch flat-panel monitor. Display resolution was set to 1024×768 pixels with a refresh rate of 60 Hz. Texts were presented in Xinhei font at 30 px, left aligned, and double-spaced. Viewing distance was adjusted to 68 cm. At this distance, each character subtended approximately 1◦ of visual angle laterally. Viewing was binocular and eye movements of the participants’ right eye were recorded using the EyeLink 1000, with a sampling rate of 500Hz.

### Procedure

**Figure 3.**
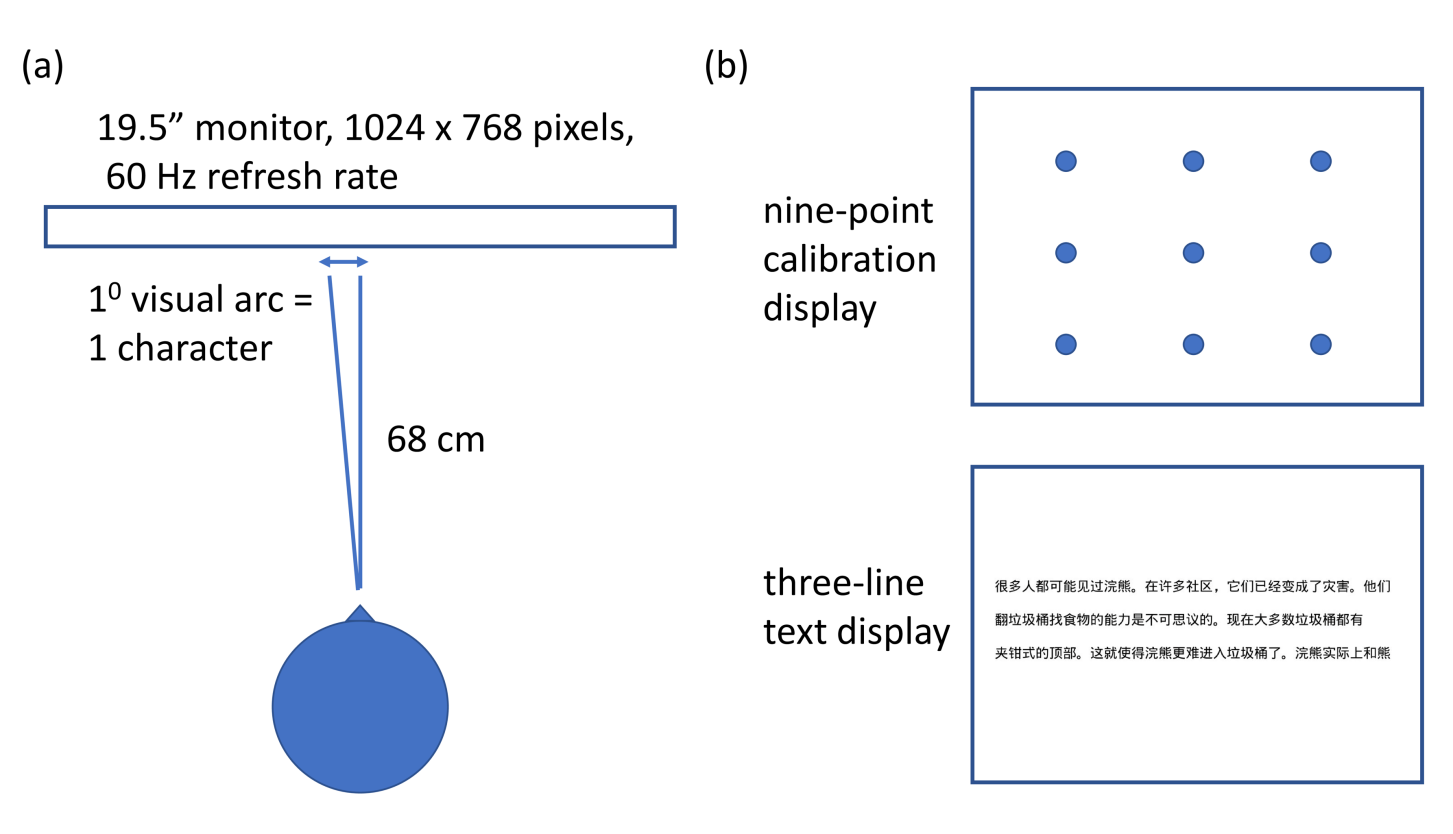
(a) A schematic representation of the experimental setup. Participants sat approximately 68 cm from the display 19.5” display. Characters subtended one degree from visual arc; (b) At the start of each reading session participants’ eye movements were calibrated using nine-point grid. Following satisfactory calibration, participants read text three lines at a time.

Participants were seated in front of the presentation monitor and received directions for the upcoming task on the display screen in front of them. Participants received identical directions for the reading task, instructing them to read every text so that they understood its meaning and were able to answer comprehension questions. They were advised to read silently. It was also explained that this was not a reading test or contest in order to make them feel more comfortable about the situation. Participants read four three-line practice trials from one story before the reading experiment, to famil-iarise them with the calibration routine and eye tracking procedures. A 9-point calibration was performed at the beginning of each story. For some participants extra calibrations were needed during the experiments due to head movements. Mean average position error in an accuracy validation routine did not exceed 0.33**^○^** of visual angle. A drift-check before every paragraph ensured accuracy between calibrations. If the drift check showed a deviation of more than 0.33**^○^** of visual angle, an additional calibration was performed. These settings (Figure 3) have proven to produce accurate and reliable data in multiple reading studies across different laboratories (25, 43). Children could take breaks between tasks or before calibrations, if necessary. Reading was self-paced and children pressed a mouse button to signal that they were done with a trial. The next paragraph or the comprehension question appeared immediately following the mouse press.

### Data pre-processing

Fixation in which participants blinked, fixations on the first and last words of each line, the first and last fixations of each trial and switch line fixations were excluded from analyses. Extreme fixation duration values less than 80 ms and of greater than 800 ms were discarded (43). Saccades located exactly at the word boundaries were also excluded. A total of 114,755 fixations contributed to the analysis. Note that the Jieba Chinese text segmentation algorithm was used for word segmentation (44). Given there is sometimes disagreement on word boundaries in Chinese, the Modern Chinese Word Dictionary was used as an arbitrator in determining the precise length of words in the experimental materials.

## Results and discussion

Linear mixed models (45) were used in the analysis of the eye movement data from the study. Statistical analysis was conducted using R 3.5.3 (46) and the lmerTest R package (47). A set of dependent measures were derived from readers’ word viewing times. As mentioned previously, the total accumulated time spent on a word during reading was partitioned into three independent components: first fixation duration (FFD), re-fixation duration (RFD), and the remainder of any viewing time on the word, re-reading duration (RRD). These components of a word’s viewing time reflect increasingly more advanced stages in the processing of the text. FFD usually reflects the initial processing of a word and tends to reflect local processing constraints. RFD is a measure of the overall difficulty of a word since it reflects the amount of additional fixations the word received before the reader moves on to the next word. RRD measures the total additional time the reader spends on the word following their initial visit and tends to reflect the difficulty a reader has in integrating a word into an overall understanding of the text. In the study, FFD is expected to reflect the immediate aspects of word processing, RFD somewhat later lexical processing, and RRD higher-level integration processes or more specifically difficulties in performing this integration (see Vorstius et al. (48) for a recent discussion of these measures).

These three components of viewing time were modelled using the same set of seven fixed independent variables and two random variables as follows.

**Figure:**
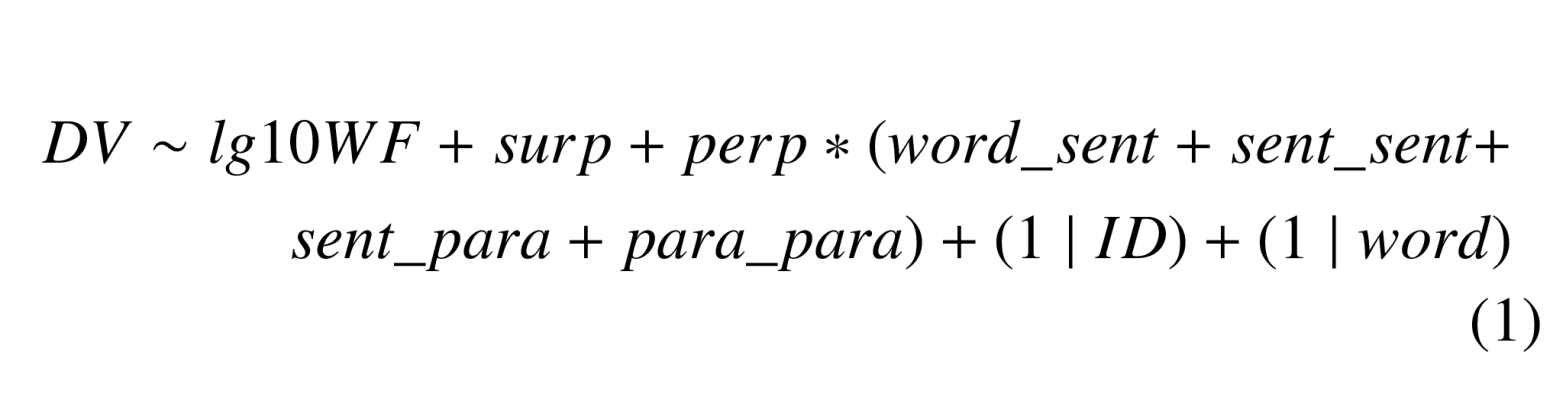


In the analysis described in detail below, the dependent variable DV could be one of the three viewing time decompositions discussed above. Participant ID and word were treated as random effects. Seven additional fixed independent variables are from four categories: word frequency, conditional probability-based surprisal, text similarity, and reading ability (a detailed description of the fixed-effect independent variables is given in Table 1).

**Table 1.**
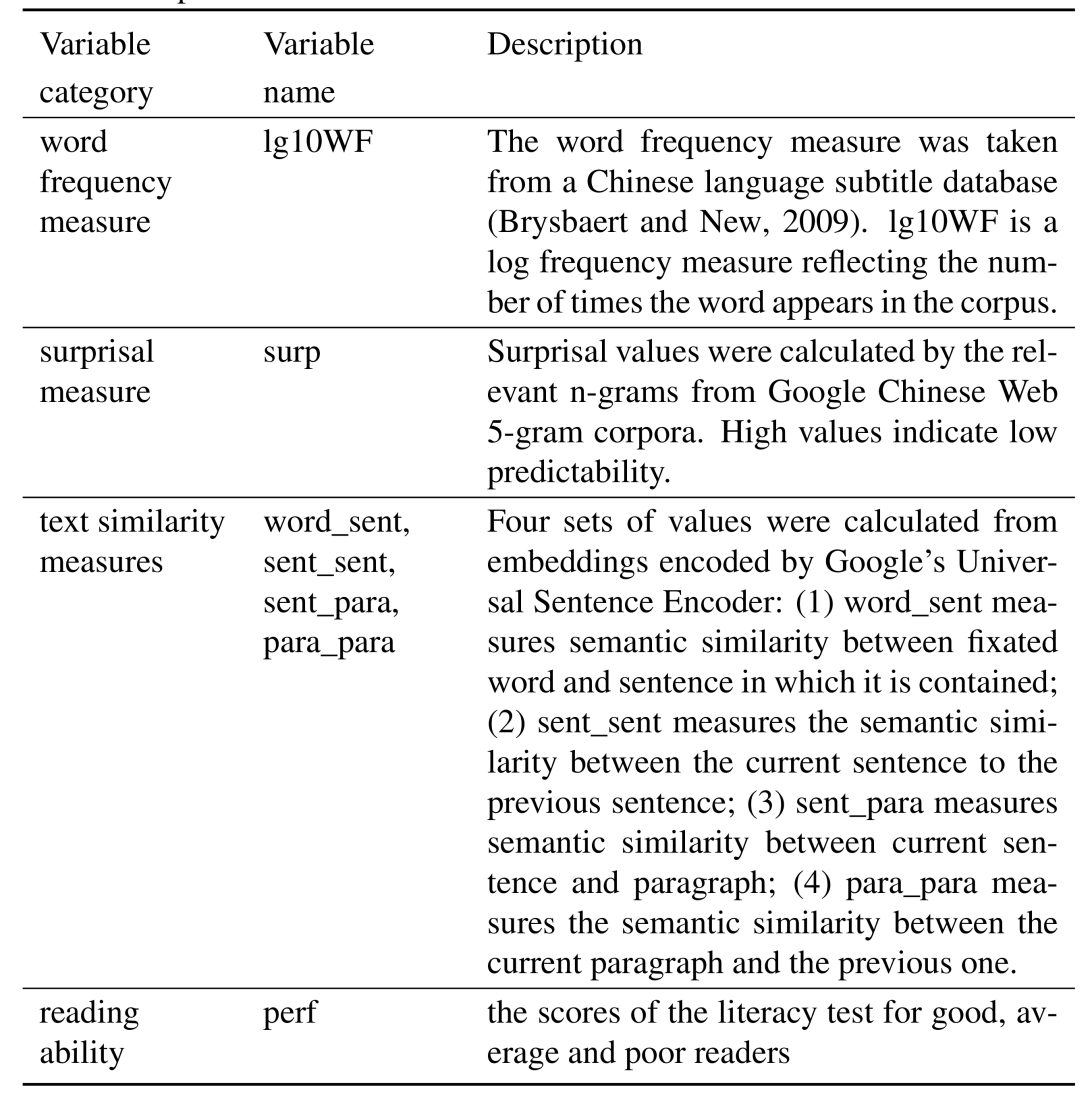
Description of the fixed-effect independent variable in the mixed-effects models

Surprisal based on n-gram derived conditional probability was calculated for each word in the texts as follows (4):

**Figure:**
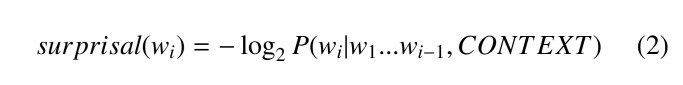


Where CONTEXT is the extra-sentential context, which will be ignored in the case of this study. P (*w_i_ |w_1_...w_i−1_*) is the conditional probability of the occurrence of *word_i_* based on its previous words. For example, the conditional probability of *word_i_* based on the previous word can be calculated by the frequency count of *word_i−1_word_i_* divided by the frequency count of *word_i−1_*. The conditional probability of *word_i_* based on the last two words can be calculated by the frequency count of *word_i−2_word_i−1_word_i_* divided by the frequency count of *word_i−2_word_i−1_*. Conditional probability based on three or four words can be similarly calculated. As mentioned in the introduction, the conditional probabilities used here were calculated using the Google Chinese Web 5-gram corpus, which consists of Chinese word n-grams and their observed frequency counts generated from over 800 million text tokens. The length of the n-grams in the corpus ranges from unigrams to 5-grams (7). In natural language processing practice, it’s more common to use trigrams where the probability of *word_i_* is conditional on the probability of its co-occurrence with the previous two words (50). Also, as n-gram length increases, the problem of data sparsity in the corpus increases. Therefore, surprisal measures are limited to those based on the previous two words in a sentence.

**Figure 4.**
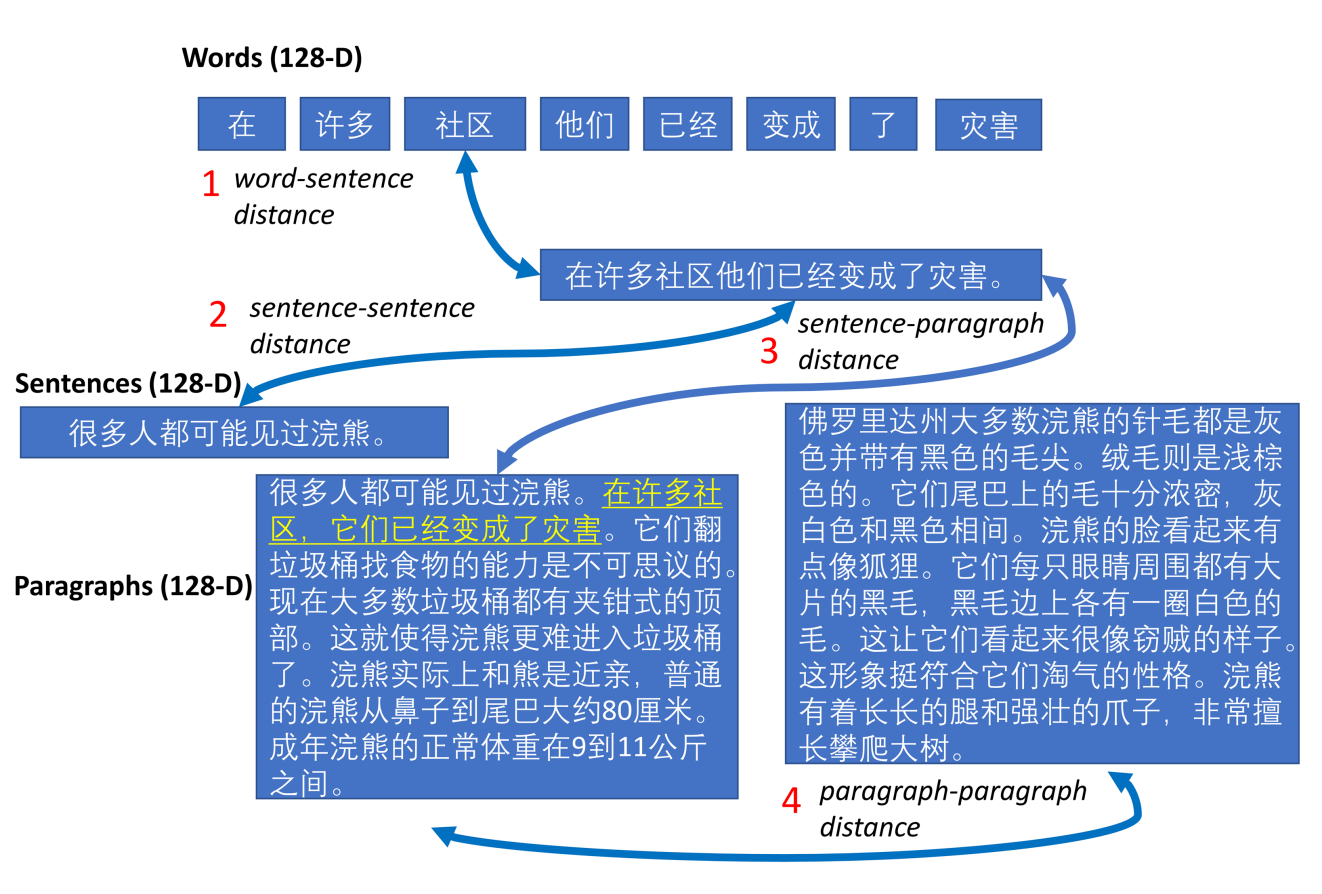
Measures of similarity at multiple scales

Four measures of text similarity (word-sentence, sentence-sentence, sentence-paragraph, paragraph-paragraph) were calculated using the embeddings derived from Google’s Universal Sentence Encoder (51). The Universal Sentence Encoder generates vectors of 128 dimensions for Chinese words, sentences, and paragraphs. The semantic similarity of pairs of language items using their embedding vectors x and y were calculated as follows (also see Figure 4):

**Figure:**
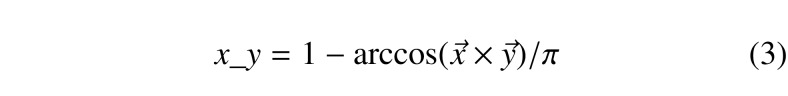


The variable word_sent measures the semantic similarity between the embeddings representing the fixated word and its containing sentence. This measure would be expected to vary significantly for each word in the sentence. For example, the similarities between function word embeddings and those of the sentence would be quite low compared to those of the sentence’s main content words. The variable sent_sent measures the semantic similarity between the sentence in which the fixated word is contained and the preceding sentence. Sentences that are similar by this measure will tend to be dealing with the same topic, whereas a topic-shift between sentences would lead to a decrease in similarity. The variable sent_para measures the semantic similarity between current sentence and its containing paragraph. The measure is intended to quantify the degree to which the current sentence is coherent with its paragraph. It can be viewed as a proxy for the potential impact on the reader of topic shifting or the introduction of new information. The variable para_para measures the semantic similarity between the current paragraph and the previous one. All four measures tap into slightly different aspects of coherence, arguably at different levels of processing. Note that all sentence and paragraph similarity measures are identical for each word in a given sentence.

**Figure 5.**
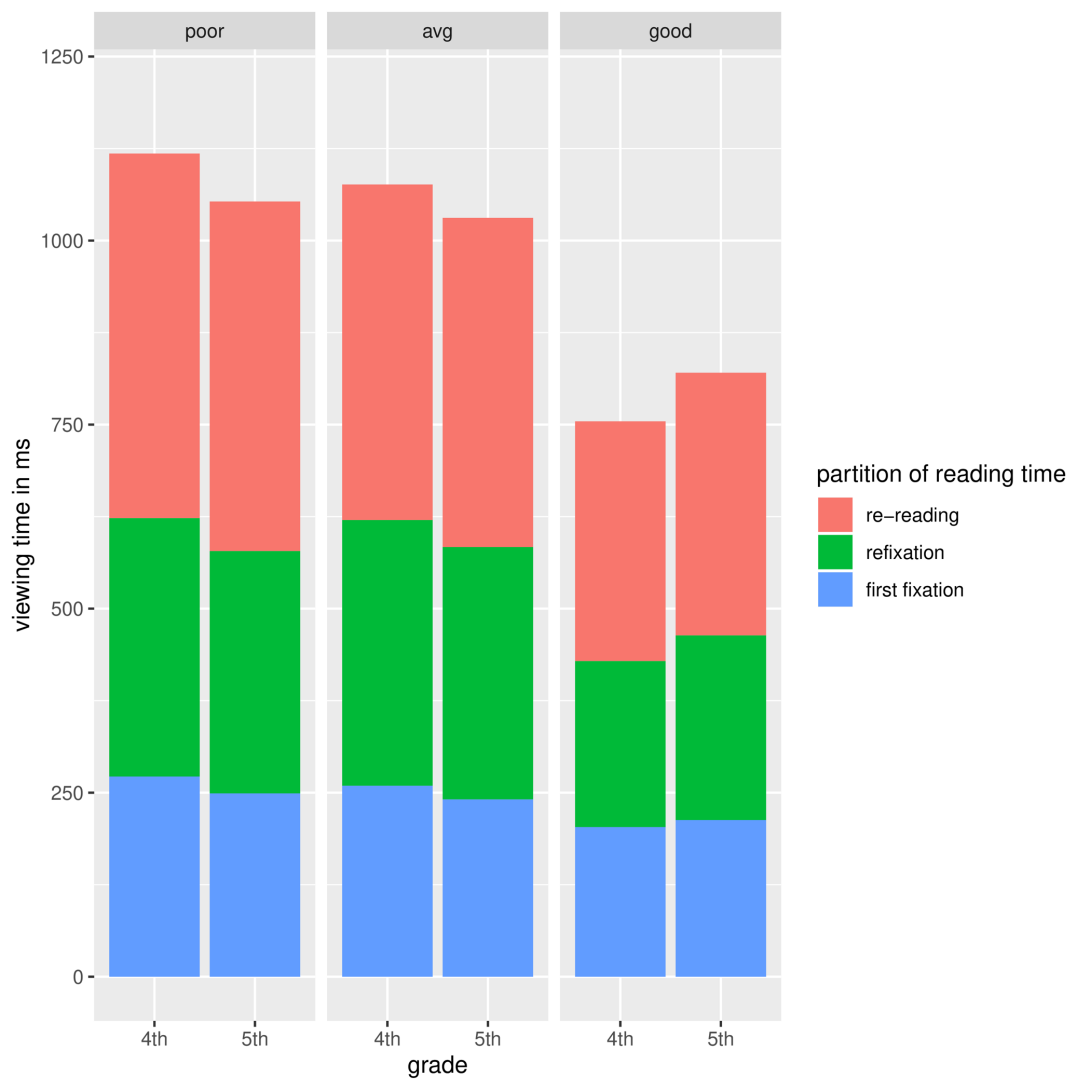
Decomposition of viewing times as a function of grade and reading ability

The means and standard deviations of the three dependent measures are summarised in Table 2. As can be seen from the table, there is a decline in the means and standard deviations of first fixation duration (FFD), re-fixation duration (RFD) and rereading duration (RRD) as readers progress from grade 4 to grade 5. Figure 5 below, shows the decomposition of viewing time as a function of reading ability and grade. We can see that the readers classified as poor and average show a slight decline in overall viewing times across grade, while those classified as good show a slight increase in overall viewing times.

**Table 2.**
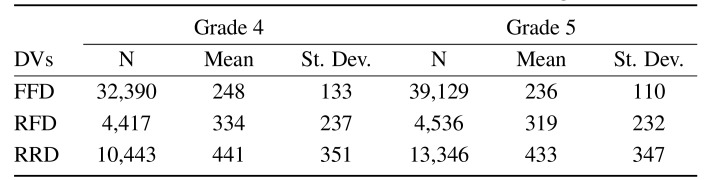
Numbers of observations, Means and SDs for the three dependent variables (DVs). Note that FFD = first fixation duration; RFD = re-fixation duration; RRD = rereading duration.

Table 3 shows the result of the mixed linear models for the three dependent measures. There were overall significant effects of word frequency for all viewing time measures: the higher the frequency, the shorter the viewing time. Reading ability had a statistically significant effect on FFD, with the more able students having shorter viewing times. Surprisal only had significant effect on FFD: the higher the surprisal, the longer the viewing time. Contrary to what was expected, the text similarity measures only had a statistically significant impact on the early viewing time (FFD): the more similar the currentand preceding sentence, the shorter the first fixation duration.

**Table 3.**
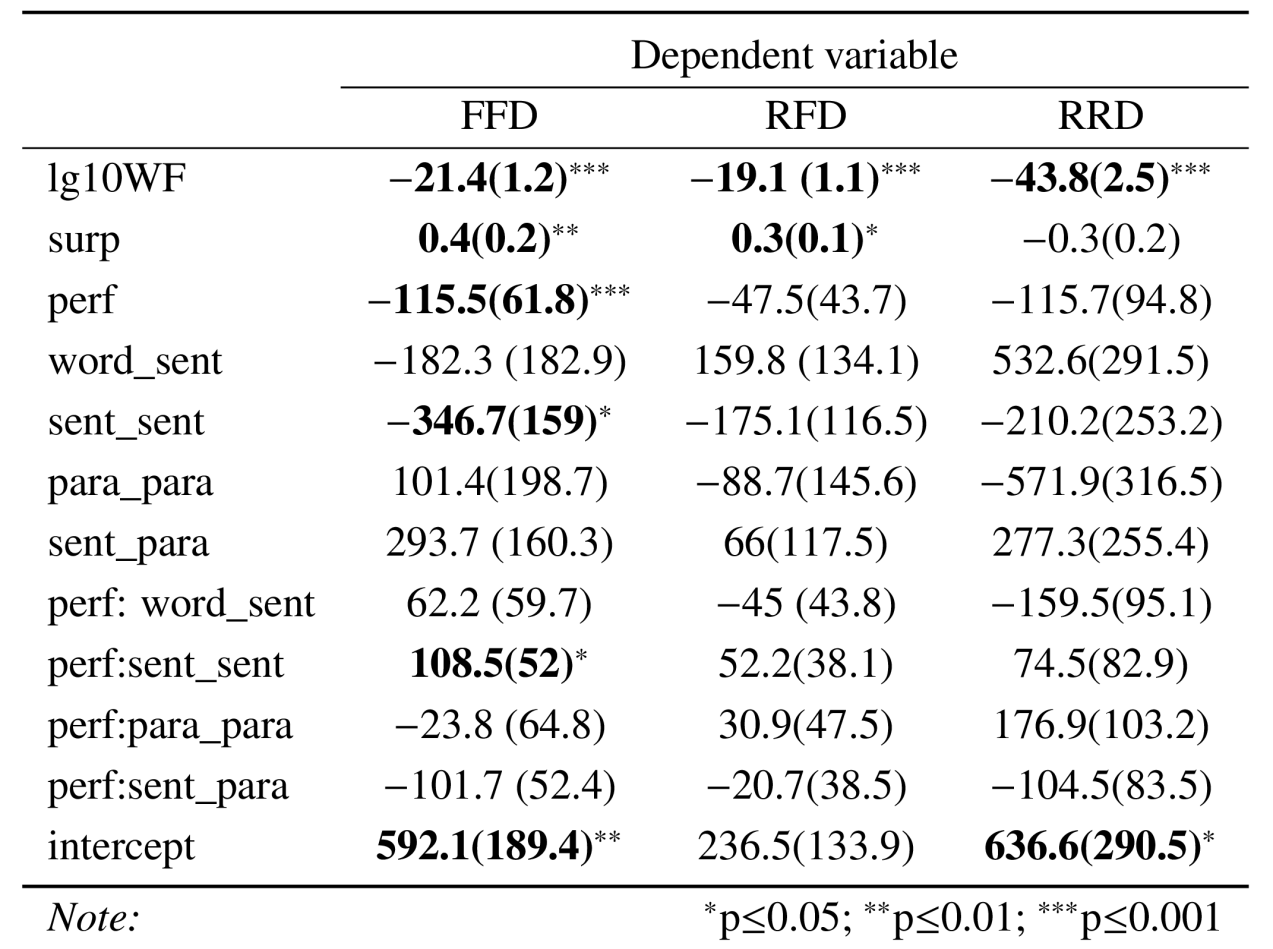
Linear mixed model estimates for the three dependent measures

However, the result of the mixed linear models for the three dependent measures done separately for each grade show a slightly different picture of FFD and RRD for fifth graders (Table 4).

### First fixation duration

**Figure 6.**
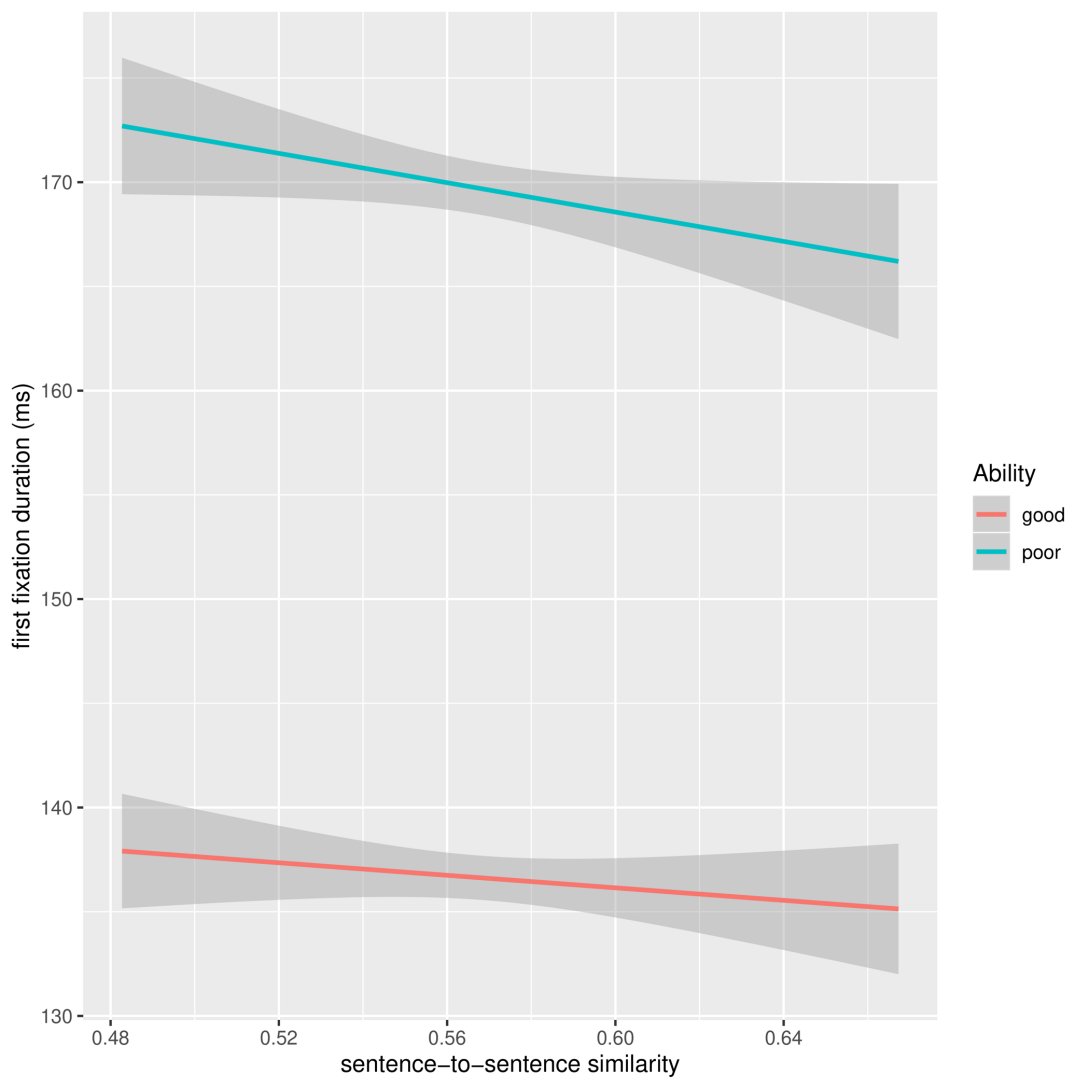
FFD as a function of current and preceding sentence similarity and reading ability: random effects removed

In the overall analysis, reading ability and word frequency were the dominant factors affecting first fixation duration (FFD) and doing so in the obvious direction - higher word frequency and greater reading ability significantly reduced FFD. Increased surprisal also significantly raised FFD - the more unexpected the word in the sentence context, the longer the FFD. One of the similarity measures, sentence-to-sentence, has a marginally significant effect on FFD, suggesting that if the current and preceding sentence are similar enough, it will shorten FFD for the words in the current sentence. However, this effect is more pronounced for poorer readers, as evidenced by the significant ability-by-similarity interaction. Figure 6 shows FFD as a function of current and preceding sentence similarity and reading ability after removal of between-subject and between-word variance of the dependent variables (52). In this figure, we can see that the source of the significant interaction is a slight floor effect, whereby there is scope for shortening the FFD as result of sentence similarity in the case of less proficient readers, but that there’s less room for improvement for the shorter FFDs of more able readers.

**Table 4.**
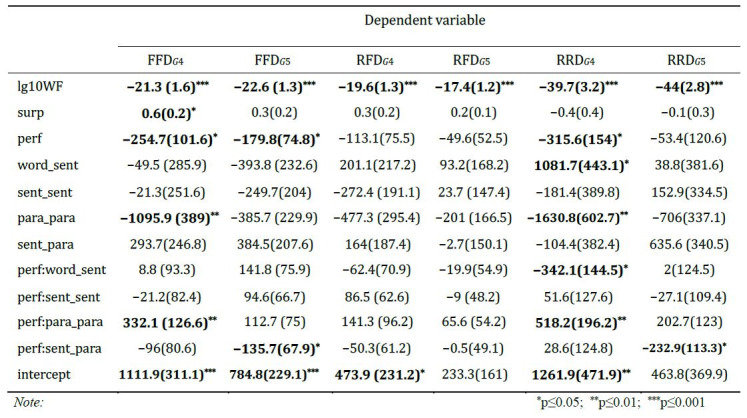
Linear mixed model estimates for the three dependent measures across grade

**Figure 7.**
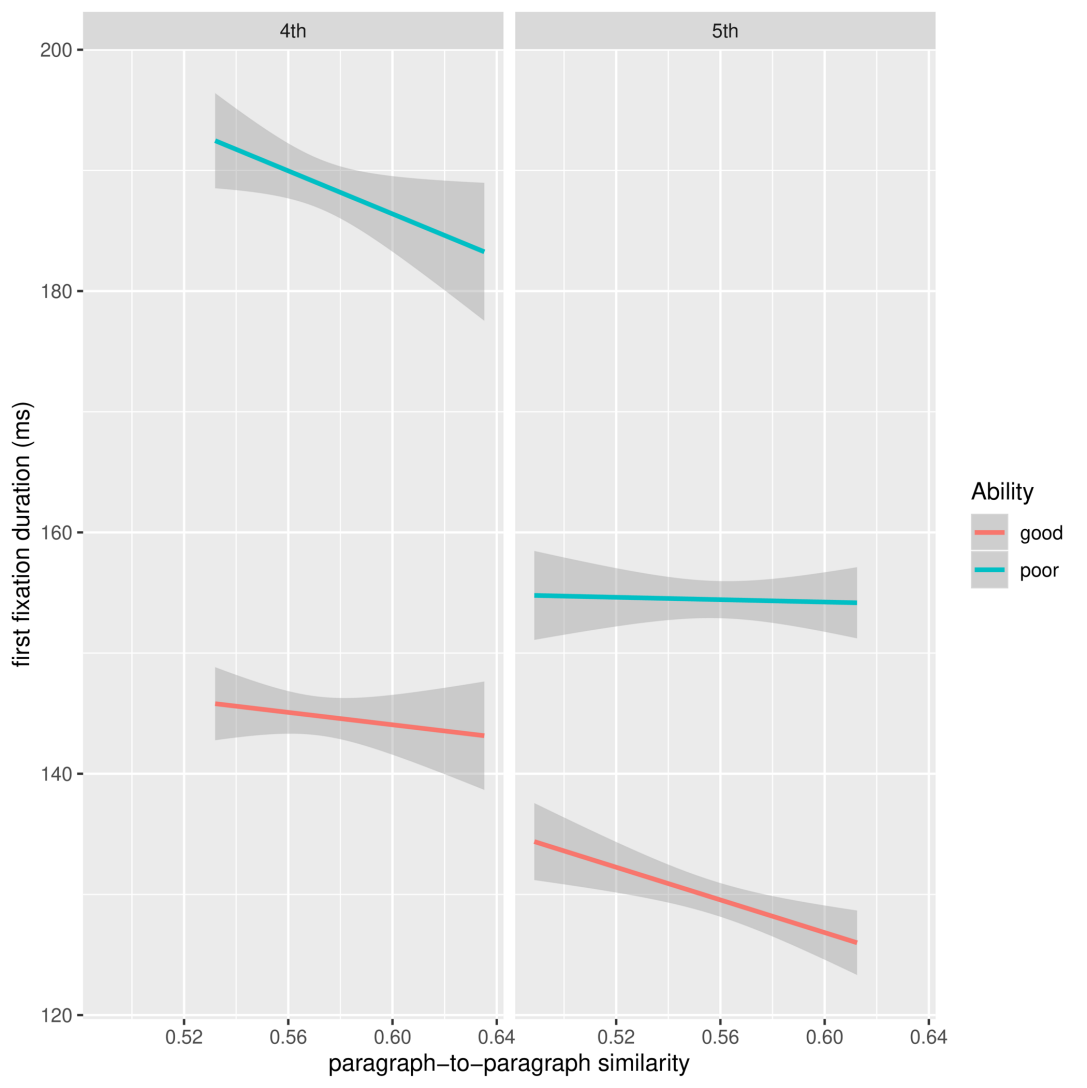
FFD for 4th and 5th grade as a function of current and preceding paragraph similarity and reading ability: random effects removed

In Table 4, grade 4 values are compared to grade 5 and apart from consistent frequency and ability effects across grades, it appears that greater paragraph similarity benefits grade 4 students more than grade 5. Moreover, reading ability also plays a role in this effect, with the less able readers benefiting more from paragraph similarity as indicated by the significant interaction with ability. This interaction is visualised in Figure 7, which shows FFD for 4th and 5th grade readers as a function of current and preceding paragraph similarity and reading ability after removal of between-subject and between-word variance of the dependent variables. The greater benefit for the less able readers may be another instance of the floor effect mentioned above, where there is an uncompressible lower bound on FFD in grade 4, which limits the amount of improvement that can be gained from exploiting paragraph similarity. The pattern of data for grade 5 in the same figure shows an overall decrease in FFD, but this time with no statistically significant interaction with reading ability.

### Refixation duration

In the global analysis of refixation duration (RFD), while there is a significant effect of word frequency and a marginal effect of surprisal, no other independent measures reach statistical significance. When we look at the analysis by grade, there is only a significant effect of word frequency. The absence of significant effects may be a consequence of the relatively small number of word refixations in the data. This probably arises from the unique characteristics of Chinese text. In the texts used for the study, 54% of the words consisted of just one character, while 42% comprised two characters. More generally, 70% of Chinese words comprise two characters, 20% one character, and 10% three or more characters. The shorter average word length in the texts reflects the fact that they were designed for reading by children. Furthermore, given the spatial compactness of the Chinese writing system, one fixation will tend to suffice in most cases for the satisfactory identification of words. Overall, therefore, the RFD measure may not be as reliable a metric of word processing in the case of Chinese reading as it is for alphabetic writing systems with more heterogeneous word lengths. In effect, the RRD measure for Chinese reading might tend to incorporate the RFD metric that normally would be distinct in alphabetic reading.

### Re-reading duration

In the global analysis, only word frequency had a significant effect on re-reading duration (RRD) and in the expected direction. No other independent measures reach overall statistical significance.

**Figure 8.**
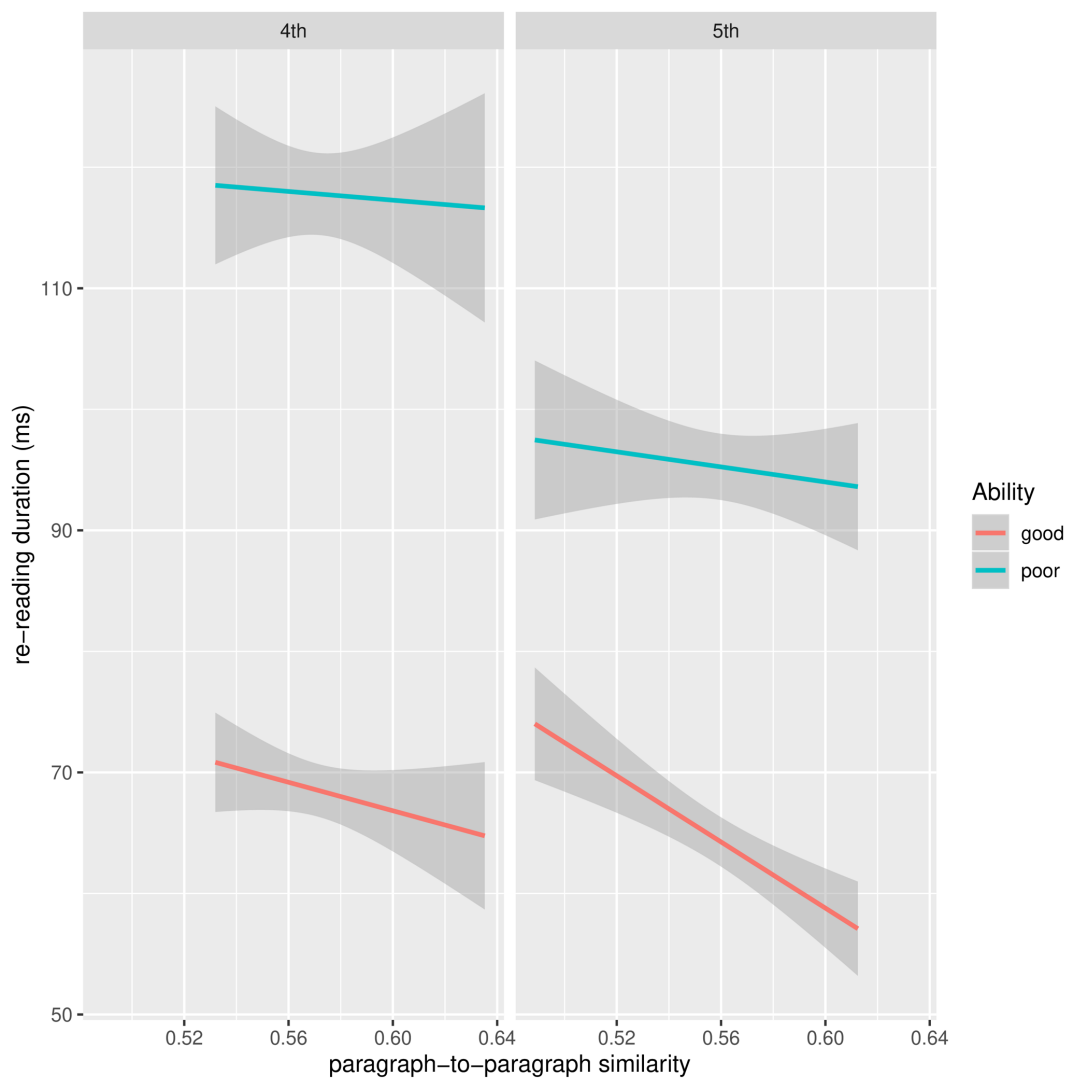
RRD for 4th and 5th grade as a function of current and preceding paragraph similarity and reading ability: random effects removed

However, when RRD is analysed separately by grade, there is a significant sensitivity to various similarity measures among grade 4 readers. The strongest effects are for paragraph similarity, with high similarity reducing RRD. Figure 8 shows RRD for 4th and 5th grade as a function of current and preceding paragraph similarity and reading ability after removal of between-subject and between-word variance in the dependent variables. The source of the interaction between ability and paragraph similarity in 4th grade readers appears this time to be the greater benefit good readers derive from paragraph similarity compared to less able readers. A similar pattern is seen for 5th grade readers, though not a statistically significant one.

**Figure 9.**
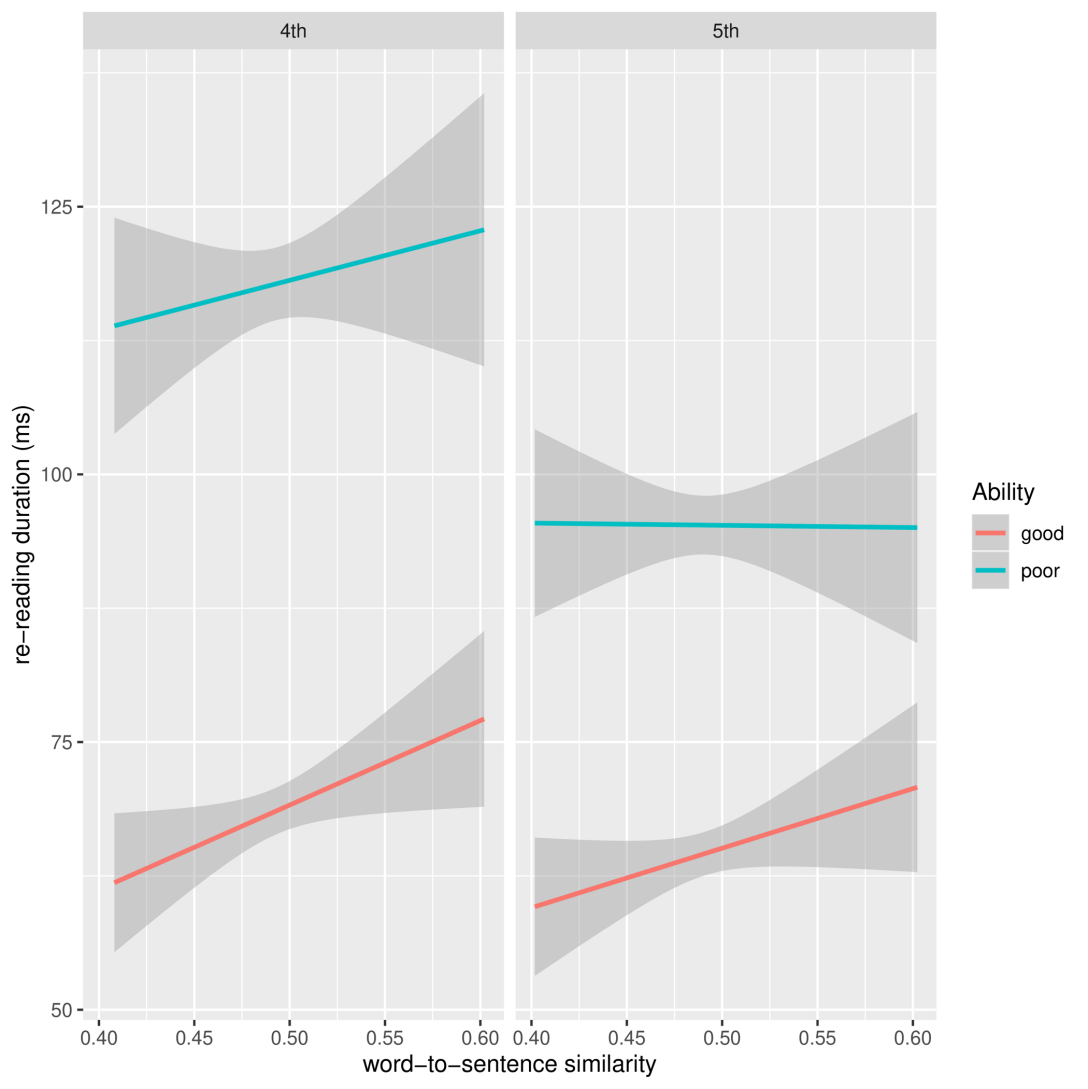
RRD for 4th and 5th grade as a function of word and sentence similarity and reading ability: random effects removed

Figure 9 shows the marginally significant interaction between reading ability and word-sentence similarity for grade 4 readers. The source of the interaction is not entirely clear and, moreover, it is not clear why greater word-sentence similarity should lead to an increase in RRD for both ability levels. One possibility is that the effect is due to the reader’s integration efforts. Recall that RRD measures repeated visits to a word, so perhaps greater similarity gives rise to an increase in confirmatory fixations. The elevated trend in RRD for good readers is also apparent in grade 5, though without reaching significance.

### Path analysis of semantic similarity measures

Given the, albeit relatively small, colinearity of the family of semantic similarity measures used in the preceding analysis and the potential complexity of interpretation of their influence as discussed above, it was decided to unpack the pattern of inter-dependence among the measures in more detail using path analysis (53). Specifically, the degree to which these measures directly and indirectly influenced two of the three dependent measures, FFD and RRD, was explored. RFD was omitted from these analyses because of the relatively low numbers of observations involved after partitioning the data and also the problematic nature of RFD given the homogeneous word length of Chinese, particularly in the children’s texts used. Another focus of the analysis was to see if the patterns of influence varied as a function of reading ability. It was hypothesised that FFD, as an early viewing time measures, would show less direct and indirect influence from the sentence and paragraph similarity measures. On the other hand, the later RRD measure was more likely to show influences of broader contextual factors. Furthermore, poorer readers should show less direct and indirect influence of extra-sentential similarities than better readers, based on the assumption that less skilled readers are more narrowly focused on local lexical context and less on the sentential and paragraph level.

**Figure 10.**
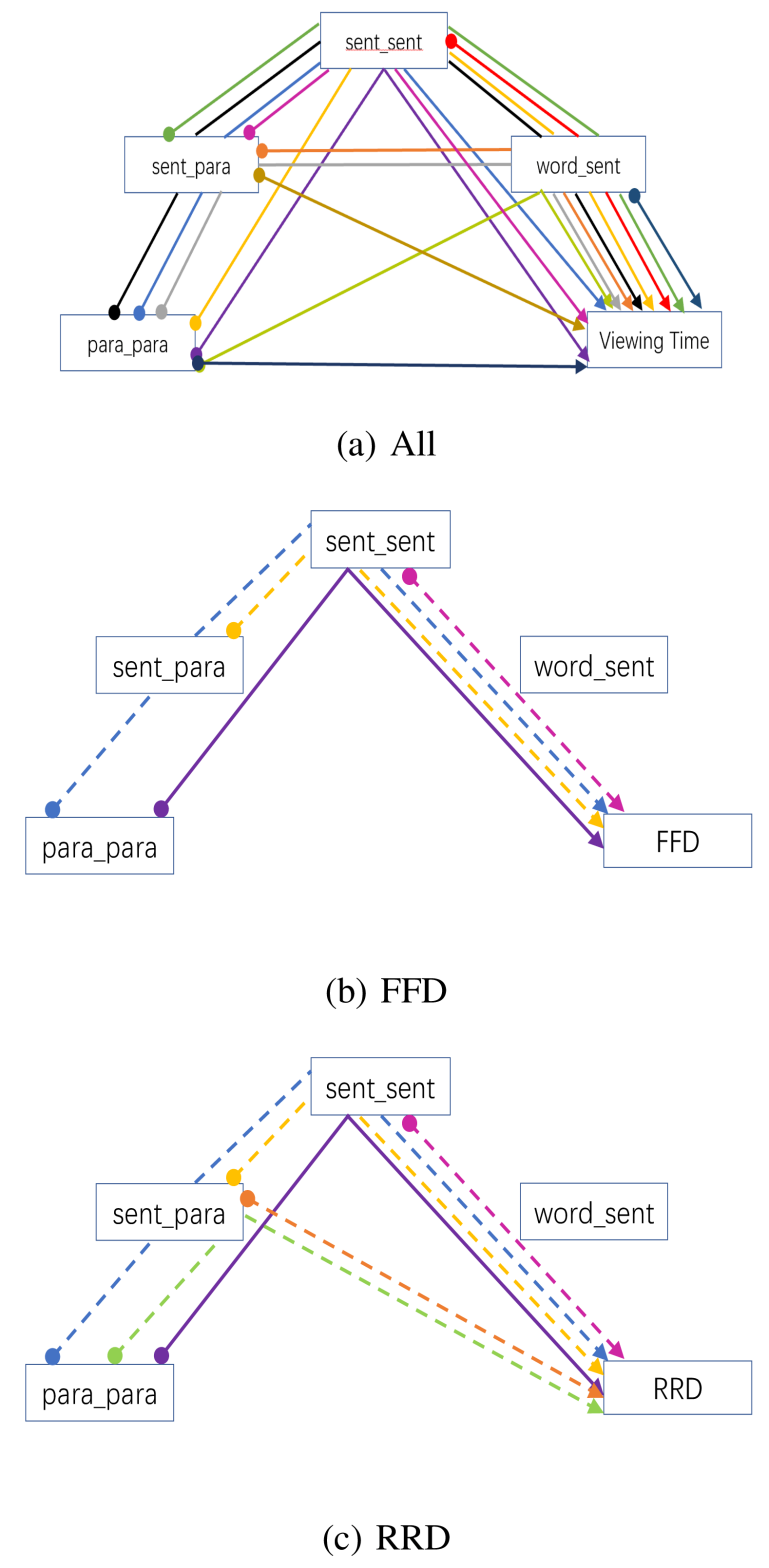
Path analysis showing the patterns of influence of the four text similarity measures used in the analysis of viewing time data: (a) all possible paths; (b) significant paths influencing first fixation duration (FFD); and (c) significant paths influencing re-reading duration (RRD). Distinct paths are illustrated with different colours, where the start of the path is represented by a filled circle and its end by an arrowhead. In (b) and (c) only paths with p < 0.05 are graphed, dashed paths indicate a negative estimate, and solid lines a positive one.

Table 5 is the output from an overall path analysis examining the pattern of influences (direct and indirect) on FFD and RRD. Examination of the pattern of significant estimates shows the significant paths for FFD are also significant for RRD, with RRD having two additional significant paths of influence from paragraph related measures. The pattern of significant paths is visualised in Figure 10, where Figure 10 (a) represents all possible paths of influence and (b) and (c) represent the paths with a significance of P < 0.05 for FFD and RRD, respectively. It is apparent that RRD shares the same pattern of influences as FFD but with additional pathways from sentence-paragraph similarity. This difference can be viewed as a progression from sentence level to paragraph level influences to which FFD and RRD are expected to be differentially sensitive. Note that the dashed paths represent negative estimates, indicating a reduction in viewing time as a function of increased similarity, whereas positive estimates indicate an increase in viewing time. Note also that word frequency and surprisal are included in the path analysis but are not shown in the path diagrams. The inclusion of surprisal explains the absence word_sentence similarity effects in this and all subsequent path analyses. When surprisal is omitted from the path analysis model, all of the paths of influence on RRD are mediated through the word_sentence variable. However, it was decided to use the full model to derive the paths in order to align better the analysis with the preceding linear mixed-model analyses.

**Table 5.**
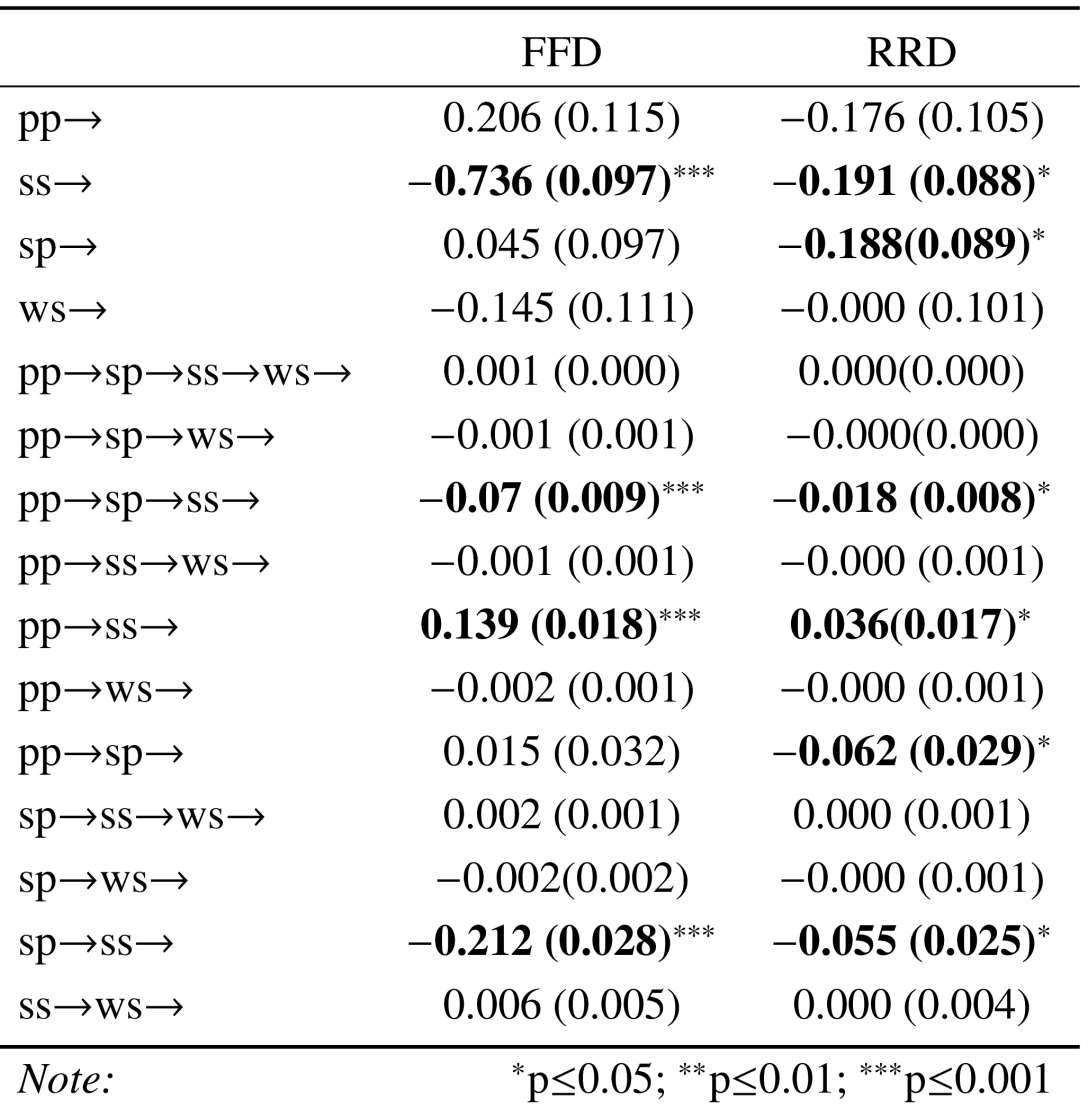
Estimates (and their standard errors) for the path analyses of the impact of the similarity measures on FFD and RRD dependent measures. Note that pp = paragraph_paragraph similarity; sp = sentence_paragraph similarity; ss = sentence_sentence similarity; ws = word_sentence similarity.

The strength of various paths’ influence on reading time measures can be considered to reflect efforts by the reader to integrate what they are reading into their developing understanding of the text. Therefore, an analysis of the strength of paths for readers of different reading ability should tell us something about readers’ growing sensitivity to higher-order properties of the text. The main measures where we would expect to see differences are in the FFD and RRD viewing times, since the preceding LMM analyses have shown that semantic similarity measures have a significant effect on these two variables.

**Figure 11.**
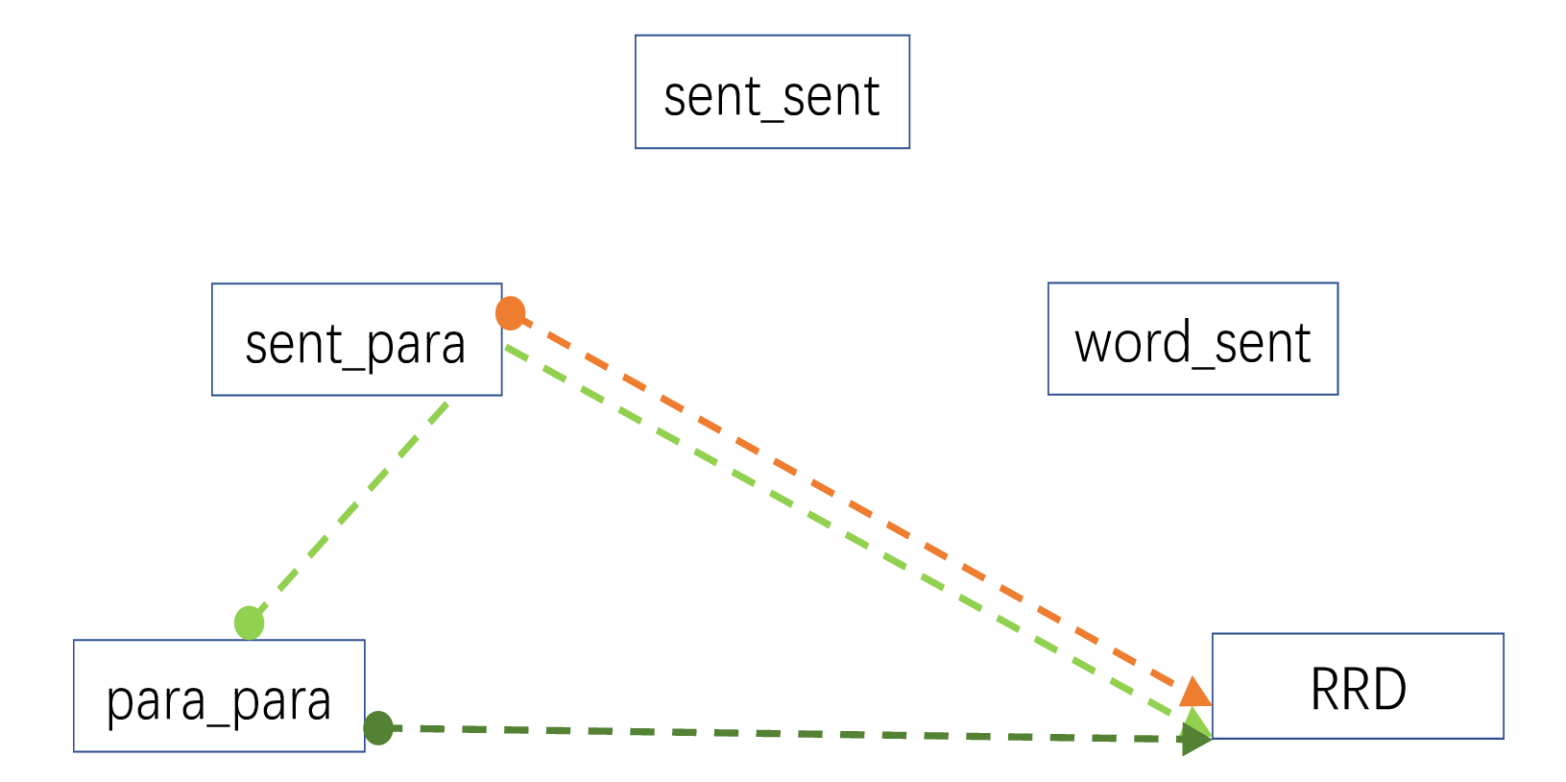
Results of a path analysis showing the significant patterns of influence (p < 0.05) of the four similarity measures used in the analysis of RRD for good readers. Distinct paths are illustrated with different colours, where the start of the path is represented by a filled circle and its end by an arrowhead. Dashed paths indicate a negative estimate and a reduction in viewing time, solid lines are positive estimates that contribute to an increase in viewing time.

Table 6 provides a partitioning of the path analysis into good and poor readers for the FFD and RRD measures. While there is little difference in the pattern of significant paths on the basis of reading ability for the FFD measure, there is a clearer divergence in the case of RRD. More able readers show viewing time benefits from greater sentence and paragraph similarity, while poorer readers show no significant benefits. The significant paths for RRD for the more able readers are illustrated in Figure 11. An equivalent figure for poorer students would show no visible paths. The one inconsistent result in these data is the significant positive estimate in Table 6 for the FFD of the less able students. It’s unclear why greater similarity of paragraphs should have an impact on FFD, let alone for less able readers and best explored further within an experimental rather than corpus-based paradigm.

**Table 6.**
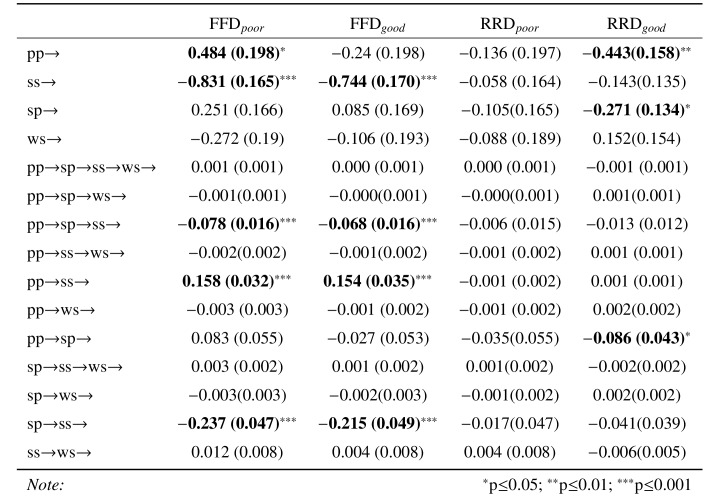
Estimates (and their standard errors) for the path analyses of the impact of the similarity measures on the first fixation duration (FFD) and re-reading duration (RRD) as a function of reading ability. Note that pp = paragraph_paragraph similarity; sp = sentence_paragraph similarity; ss = sentence_sentence similarity; ws = word_sentence similarity.

Overall, the path analysis results have highlighted the potential for using the emergence of sensitivity to high-order similarity measures (e.g., sentence and paragraph similarity) as indices of reading development. Alternatively, a lack of such sensitivity may serve as an indicator of readers at risk. It is important to note that the current study was a corpus-based analysis, using naturally occurring patterns of word, sentence, and paragraph similarity and cohesion. The next step is to design controlled experiments where we probe the finer detail of the sensitivity uncovered in this preliminary exploration. Such experiments would involve, for example, controlling the specific text features that serve to increase or decrease similarity. In addition, there may be identifiable sub-groups of readers who vary in their sensitivity to specific similarity configurations.

## Conclusion

The results described in this paper demonstrate that text similarity measures have a significant impact on moment-to-moment processing of words in reading. Previous research has demonstrated that n-gram and LSA contextual measures have an impact on word viewing times in adult readers (8, 9). However, this is the first attempt to track the developmental trajectory of these influences in Chinese early readers as well as readers with differing reading abilities.

While the underlying factors driving the developmental change in response to supra-lexical properties of the text clearly need to be explored further, on the basis of what has been found one should be cautious about attributing the main changes in the developing reader merely to changes in the efficiency of lexical processing (54, 55, 56, 57). The study finds that there are robust contextual effects impacting on the local processing of words during a fixation and the nature of these effects changes as the reader becomes more proficient.

Another contribution this paper is to present an easy-to-use set of tools for linking the low-level aspects of fixation durations to a hierarchy of sentence-level and paragraph level features that can be computed automatically. The use of the decomposition of word viewing times into immediate and later components combined with measures of sentence and paragraph coherence illuminates the time-course of the reader’s processing of a text. Similar to the study by Radach et al. (58), broader contextual constraints have been shown to impact on low-level aspects of the reading process.

Finally, the similarity-based measures could also be used to assess text for their suitability for readers of different levels of ability. While there are text complexity measures such as “Lexile” (59) available for English texts, nothing comparable exists for Chinese. The measures described here could be applied to any language for which there is a large text corpus. Moreover, the Lexile measure is primarily calculated as a function of word frequency and sentence length. The text coherence measures described here could usefully augment a lexile-like measure to provide quantitative measures of the semantic characteristics of the sentences and texts in addition to word frequency and sentence length.

### Ethics and Conflict of Interest

The authors declare that the contents of the article are in agreement with the ethics described in http://biblio.unibe.ch/portale/elibrary/BOP/jemr/ethics.html and that there is no conflict of interest regarding the publication of this paper.
